# Emerging role of HMGB1 in fibrotic diseases

**DOI:** 10.1111/jcmm.12419

**Published:** 2014-10-06

**Authors:** Liu-Cheng Li, Jian Gao, Jun Li

**Affiliations:** a Anhui Key Laboratory of Bioactivity of Natural Products, School of Pharmacy, Anhui Medical UniversityHefei, China; b Third-Grade Pharmaceutical Chemistry Laboratory of State Administration of Traditional Chinese Medicine (TCM-2009-202), Pharmaceutical Preparation Section, The First Affiliated Hospital of Anhui Medical UniversityHefei, China

**Keywords:** high-mobility group box 1, cystic fibrosis, liver fibrosis, renal fibrosis, pulmonary fibrosis, myocardial fibrosis

## Abstract

High-mobility group box 1 (HMGB1) is originally identified as a DNA-binding protein that functions as a structural co-factor critical for proper transcriptional regulation in somatic cells. Recent studies indicate that HMGB1 can be passively released from necrotic cells or actively secreted into the extracellular milieu under appropriate signal stimulation. Extracellular HMGB1 is a multifunctional cytokine that contributes to the process of infection, injury, inflammation, apoptosis, and immune responses by binding to specific cell-surface receptors. Recently, emerging studies indicate that HMGB1 is closely involved in fibrotic disorders including cystic fibrosis, liver fibrosis and pulmonary fibrosis, while HMGB1 signal inhibitions protect against the experimental models of fibrotic diseases. From a clinical perspective, HMGB1 represents a current challenge that can be exploited orchestrate reparative responses. This review focuses on the crucial role of HMGB1 in the pathogenesis of fibrotic diseases and inhibition of which may represent a promising clinical approach for treating tissue fibrosis.

- Introduction
- Structure and functions of HMGB1
  – Structure of HMGB1
  – Receptors of HMGB1
  – Functions of HMGB1
- HMGB1 in fibrotic disorders
  – Systemic sclerosis
  – Cystic fibrosis
  – Liver fibrosis
  – Renal fibrosis
  – Pulmonary fibrosis
  – Myocardial fibrosis
- Targeting HMGB1 as therapy in fibrotic diseases
- Conclusions and future prospect

## Introduction

Tissue fibrosis is characterized by abnormal accumulation of extracellular matrix (ECM) molecules that make up excessive tissue scarring and promote chronic organ injury, occurs in a variety of physiological systems including lung, liver, kidney and heart [1]. Nevertheless, the pathological fibrosis among multiple organs share the core features including epithelial injury and dysfunction, appearance of myofibroblasts, ECM accumulation, immune cell recruitment, monocyte-derived cells, and the resolution and regression of fibrosis, resulting in progressive scarring and loss of organ functions [1,2]. According to statistics, as much as 45% of deaths in the developed world can be ascribed to fibrotic diseases such as interstitial pulmonary fibrosis (PF), liver fibrosis and progressive renal fibrosis [1,3]. In recent years, a large amount of researches have put into the field of tissue fibrosis and several polypeptide mediators have been found central to the fibrotic process. However, there is currently limited effective treatment for fibrotic diseases. As the severe scarring of tissues and the accompanied end-stage fibrosis are thought to be irreversible in most cases. Therefore, understanding the novel factors which are aberrantly expressed in the animal models of fibrosis and fibrotic patients is paramount and promising to develop the therapeutic targets and treatment strategies for fibrotic diseases.

High-mobility group box 1 (HMGB1) is a highly conserved DNA-shepherding protein that is abundant in cell nucleus. It can also translocate to the cytoplasm as well as the extracellular space during cell activation, injury or death [4]. On the one hand, HMGB1 can be actively secreted from multiple cell types including macrophages, monocytes, natural killer cells, dendritic cells (DCs), endothelial cells and platelets [4]. On the other hand, it can also be passively released from necrotic or damaged cells. Both ways can discharge significant amounts of extracellular HMGB1 [5]. However, the biological activity of HMGB1 depends on its location, context and post-translational modification. Through engagement with its cell-surface receptors on immune cells, HMGB1 activates intracellular cascades that regulate several cell functions, including inflammation, chemotaxis, and microvascular rolling and adhesion [6–9].

Recently, emerging findings indicate that HMGB1 is abnormally increased in fibrotic patients and activated in fibrotic models, while HMGB1-related inhibitions protect against fibrosis, suggesting that HMGB1 may be an important mediator in fibrosis as well as a successful therapeutic strategy for the treatment from a clinical perspective. Therefore, we focus here on and briefly summarize the structure and functions of HMGB1, the crucial role in tissue fibrosis, as well as a promising potential target in fibrogenesis.

## Structure and functions of HMGB1

### Structure of HMGB1

High-mobility group box 1, one member of the HMGB family, was originally described as a nuclear non-histone DNA-binding protein that functions as a structural co-factor critical for proper transcriptional regulation in somatic cells [10]. The HMGB family contains HMGB1, -2, and -3 [11], among which HMGB1 is the most abundant non-histone nuclear protein, and it is to some degree also cytoplasmically expressed as it shuttles back and forth from the nucleus [7].

**Fig. 1:**
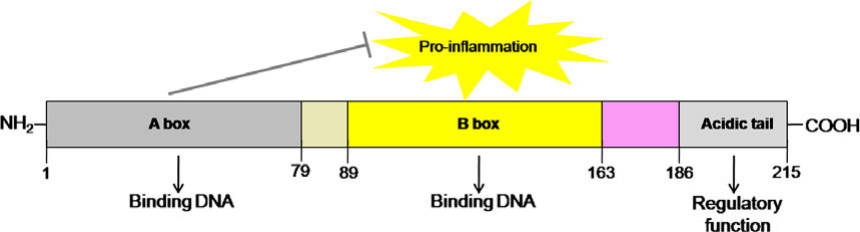
Structure and functions of HMGB1. HMGB1 has 215 residues organized into two DNA-binding domains, A box and B box, and a negatively charged C-terminus that contains a string of glutamic and aspartic acids [12,14]. The HMGB1 B box exhibits pro-inflammatory activities, in addition to DNA binding, whereas the A box alone acts as a specific HMGB1 antagonist [7,17]. The acidic tail of HMGB1 is involved in regulating DNA binding and DNA damage repair [18,19].

The structure of human HMGB1, a 25-kD protein of 215 amino acids, organized into two positively charged DNA-binding domains, homologous regions ∼80 amino acids long, termed the A box and B box, and a negatively charged C-terminus composed of 30 glutamic and aspartic acids, exclusively [8,12–14] (Fig. 1). It has been reported that the primary HMGB1 sequence is 98.5% identical in all mammals, and two of the three substitutions occur in the repetitive carboxyl terminus with switches of aspartic and glutamic acids [8]. In addition, the A box and B box are helical structures, partly covered by the acid tail, are involved in DNA double chain folding and distortion [13,15]. They are similar in conformation despite their limited amino acid identity [16]. Structure-functional studies of the full-length HMGB1 have revealed that the extracellular cytokine activities such as the pro-inflammatory effect, resides within the B box. However, the cytokine role of B box can be competitively inhibited by the specific HMGB1 antagonist, truncated A box protein [7,8,17]. In addition, the C-terminal acidic tail is suggested to play an indispensable role in regulating DNA binding and DNA damage repair, and responsible for the inhibitory effects of HMGB1 on efferocytosis [18–20].

### Receptors of HMGB1

Like many other nuclear cofactors, HMGB1 is later discovered to have another role as an intercellular messenger molecule, released from a variety of cells into the extracellular milieu to act on specific cell-surface receptors. Emerging researches indicate that HMGB1 has a broad repertoire of immunological activities such as induction of cytokine production, cell proliferation, chemotaxis and differentiation as well as the modulatory activities of haematopoietic, epithelial and neuronal cells and systemic effects [4,8]. These activities reflect the function of HMGB1 as an alarmin and the signal transduction is closely associated with toll-like receptor 2 (TLR2), TLR4, TLR9, the receptor for advanced glycation end products (RAGE), and CD24-Siglec-10 [6,21–24], while the equivalent amount of HMGB1 alone has no such effect [25]. Apart from a direct receptor interaction, HMGB1 may form heterocomplexes with other molecules, such as interleukin (IL)-1, CXC chemokine ligand 12 (CXCL12), DNA, RNA, histones or lipopolysaccharide (LPS), which generate synergistic responses compared with those produced by the individual components [7]. The signal is mediated by acting on its own or in heterocomplex formation, and then initiates chemotactic activity and release of pro-inflammatory cytokines, leads to epithelial barrier dysfunction, and acute and chronic inflammation.

RAGE is a multi-ligand receptor that belongs to the immunoglobulin superfamily of transmembrane proteins that is located in the vicinity of the MHC complex in humans and mice [26]. In response to inflammation, the expression of RAGE is dramatically induced in type I alveolar epithelial cells (AECI) and infiltrated inflammatory cells, suggesting that RAGE might have an important role in lung pathophysiology [27]. Signalling through RAGE contributes to HMGB1-induced secretion of pro-inflammatory cytokines, which is reduced by 70% in RAGE knockout mice [28]. As this effect was not complete, other receptors such as TLR2 and TLR4 also appear to be involved in HMGB1 signalling. For cytokine induced by HMGB1, TLR4 signalling is strictly required. Surface plasmon resonance studies indicate that HMGB1 binds specifically to TLR4, and that this binding requires a cysteine in position 106. Moreover, inhibition of TLR4 binding with neutralizing anti-HMGB1 monoclonal antibody (mAb) or by mutating cysteine 106 prevents HMGB1 activation of pro-inflammatory cytokines [29].

### Functions of HMGB1

HMGB1 has multiple functions in infection, organ dysfunction, inflammation and immune responses [8]. In the nucleus, HMGB1 acts as a nuclear DNA chaperone that takes part in DNA replication, transcription, recombination, repair, as well as in chromatin stability and in regulating the transcriptional activity of steroid hormone receptors and glucocorticoid receptors, through exerting effects on chromosomal architecture [13,30–32]. However, with cell activation, injury or death, HMGB1 can translocate outside of the cell [4]. Extracellular HMGB1 has become a focus on eliciting and promoting the inherent immunity including neurite outgrowth, platelet activation, and cytokine- and chemokine-like activity through binding to various receptors on the surface of responding cells [7,15]. It targets multiple immunologically relevant systems including p53, nuclear factor (NF)-κB and the glucocorticoid receptor [33–35]. There, HMGB1 can serve as a damage-associated molecular pattern or alarmin to stimulate the innate immune system either by itself or as part of complexes with cytokines, as well as other exogenous and endogenous molecules [8].

The capacity for HMGB1 to undergo multiple molecular contacts might thus be essential for the protein to act as both an intracellular and extracellular mediator. Consistent with an important role for its transcription, HMGB1 deficiency is demonstrated lethal. Previous study has shown that HMGB1 knockout mice dies within 24 hrs because of hypoglycaemia after birth, while cell lines lacking HMGB1 grow normally *in vitro*, indicating that HMGB1 is not necessary for the overall organization of chromatin in the cell nucleus, but is essential for proper transcriptional control by specific transcription factors [36]. Recombinant B box causes nuclear translocation and activation of NF-κB family members, p65 and p50, in human myeloid DCs [37]. Endogenous HMGB1 secreted by LPS-stimulated DCs contributes to late but not early activation of extracellular signal-regulated kinase 1/2 (ERK1/2), NF-κB and to a smaller extent p38 mitogen-activated protein kinase (MAPK) [38]. The ability of necrotic cells to maximally stimulate NF-κB activity in macrophages is at least in part dependent on a TLR-mediated signalling pathway, and both TLR2 and TLR4 have been implicated in the activation of macrophages by HMGB1 [21]. TNF-α secretion from HMGB1-stimulated mouse macrophages is not altered in TLR2-knockout mice [28]. In skin tumourigenesis, TLR4-dependent inflammatory response that leads to tumour development is dependent on HMGB1 [39]. Further evidence reveals that the signalling of HMGB1 or with its receptors plays an essential role in mediating fibrotic diseases in liver, renal, lung and myocardial (Table 1).

**Table 1:** The role of HMGB1 in fibrotic diseases, with focus on the experimental cases, samples, related receptors and regulatory role

	Samples	Related receptors	Regulatory role	References
Cystic fibrosis				
Patients	Sputum, serum, and BALF	C-X-C chemokine receptor	Induce matrix degradation and neutrophil influx, predict incidence and recurrence, impair phagocytosis	[39,53,54]
Scnn1b-Tg or CFTR^−/−^ mice	BALF	TLR4	Neutrophil influx, bacterial infection, impair phagocytosis	[53,54]
Liver fibrosis				
HSC(-T6) cells	Cells, supernatant	–	Increase α-SMA expression and collagen synthesis, suppress matrix metalloproteinases-2 activity	[59,61]
Patients	Serum, human primary HSCs	TLR4	Distinguish fibrotic degree, induce proliferation and migration, promote fibrosis	[60,63]
Sprague–Dawley rats	Liver tissues, serum	TLR2, TLR4	Correlate with collagen deposition, pro-inflammatory mediators	[61,62]
Renal fibrosis				
Proximal tubular epithelial cells	Cells	RAGE	Induce EMT *via* RAGE and TGF-β1	[67]
Pulmonary fibrosis				
C57BL/6, RAGE^+/−^, or RAGE^−/−^ mice	BALF, lung tissues, AECII, primary AEC	RAGE	Induce EMT, injury, TGF-β1 and PDGF production	[70,71]
Patients	Serum, BALF, lung tissues, washing medium	RAGE	Inflammation, apoptosis, and fibrosis	[72,74,75]
WI-38 lung fibroblasts, primary rat and human AEC	Cells, supernatant	–	Induce proliferation, increase wound closure	[72,73]
Myocardial fibrosis				
C57BL/6J mice	Hearts, cardiac fibroblasts	–	Dependent on MAPK signalling	[78]

BALF, bronchoalveolar lavage fluid; TLR, toll-like receptor; HSC, hepatic stellate cell; α-SMA, alpha-smooth muscle actin; RAGE, receptor for advanced glycation end products; EMT, epithelial–mesenchymal transition; TGF-β, transforming growth factor-beta; AEC, alveolar epithelial cells; PDGF, platelet-derived growth factor.

## HMGB1 in fibrotic disorders

### Systemic sclerosis

Progressive fibrosis, which distorts tissue architecture and results in progressive loss of organ function, is now recognized to be one of the major causes of morbidity and mortality in patients with systemic sclerosis (SSc) [40]. SSc is a complex connective tissue disease characterized by fibrosis of the skin and various internal organs including heart, lung and renal [41]. It has been reported that endothelium and pericyte activation, telocytes loss, aberrant immune responses, endoplasmic reticulum stress and chronic tissue injury are involved in the initiation of fibrosis in SSc [40–42]. Current molecular targets of SSc endothelium dysregulation are endothelin−1, platelet−derived growth factor (PDGF) signalling, 5−hydroxytryptamine and VEGF [40], while HMGB1 may be also a potential target for SSc from the collected data.

Early reports have demonstrated that antibodies to HMGB1 and HMGB2 are found in about 1/3 of SSc Sera and anti-HMGB1/HMGB2 antibodies are detected commonly in systemic rheumatic diseases, particularly in rheumatoid arthritis and SSc [43,44]. Another study has shown that serum HMGB1 level in SSc is higher than that in healthy controls and control mice, while SSc patients with elevated HMGB1 level have more frequent involvement of several organs and immunological abnormalities compared to those with normal level [45]. Furthermore, the HMGB1 level correlates positively with modified Rodnan total skin thickness score and negatively with pulmonary function test [45]. These results suggest that elevated serum HMGB1 is associated with the disease severity and immunological abnormalities in SSc. Further studies have revealed that platelet HMGB1 depletion is significantly associated in SSc patients with degranulation and with expression of P-selectin as well as with fibrinogen binding to their plasma membrane [46]. In addition, the bioactive HMGB1 from activated platelets can stimulate neutrophils to generate reactive oxygen species *via* P-selectin, which significantly increase the ability of extracellular HMGB1 to activate blood leucocytes [47]. These findings indicate that platelets represent a source of HMGB1, in the vasculature of SSc patients, possible contributing to endothelial cell activation and persistent microvascular injury.

However, it is noteworthy that telocytes, a distinct stromal cell population other than fibroblasts, fibrocytes, fibroblast-like cells and mesenchymal cells, are severely damaged and progressively disappear from skin lesions in patients with SSc [42,48]. In addition, telocytes loss contributes to altered skin homoeostasis and 3D organization of the ECM in SSc skin, as well as impaired skin regeneration and diminished functional stem cell niches [41,42,49]. A recent study has demonstrated that extracellular HMGB1 level influences the quality of healing in cutaneous wounds [50]. It suggests that HMGB1 may play a role in SSc skin and other organs, and the activation of HMGB1 may be associated with the loss of telocytes, which are involved in intercellular signalling that can influence the transcriptional activity of neighbouring cells and may be attractive novel cells in fibrotic diseases [40,51].

### Cystic fibrosis

Cystic fibrosis (CF) is the most common lethal genetic disorder among Caucasians, but disease occurs worldwide. Approximately, 10 million Americans carry mutations, while 25,000 suffer actual disease [52]. CF is characterized by an unrelenting neutrophil-predominant airway inflammatory response which leads to ECM remodelling and eventually to the development of bronchiectasis.

Recent data suggest that HMGB1 may play an important and underappreciated role in inflammation. Early finding has shown that the increased expression of HMGB1 in samples that derived from patients with CF and from a murine model of CF lung disease (Scnn1b-transgenic [Scnn1b-Tg] mouse) is directly chemotactic for neutrophils through a C-X-C chemokine receptor (CXCR)-dependent mechanism, while intratracheal instillation of HMGB1 in mice triggers neutrophil influx and contributes to lung matrix degradation [53]. Moreover, intratracheal injection of wild-type mice with recombinant HMGB1 results in neutrophilic influx and resultant production of proline–glycine–proline, as also observed in the airway secretions of CF cases. Further researches have demonstrated that the elevated levels of HMGB1 in *Pseudomonas aeruginosa*-induced CF mice are significantly reduced even with the diminished neutrophil infiltration and alveolar injury, and increased clearance of *P. aeruginosa* in the lung by administration of specific neutralizing anti-HMGB1 mAb [54]. In addition, the HMGB1-mediated suppression of bacterial phagocytosis is attenuated in macrophages lacking TLR-4, suggesting a critical role for TLR4 in signalling HMGB1-mediated macrophage dysfunction [54]. It reveals a novel role for HMGB1 in host defence by both mediating neutrophil infiltration and attenuating bacterial clearance in *P. aeruginosa* pneumonia. In CF sputum, high HMGB1 level has the potential to reflect concurrent clinical status and predict future outcome of acute pulmonary exacerbations and survival, which is plausibly because it mediates long-term airway inflammation [52].

Current findings support the pro-inflammatory effects of HMGB1 in the CF airway and provide potentially useful new measurements for monitoring short and longer term treatment effects for CF. Nevertheless, further studies are needed to clarify the relative contributions that HMGB1 or with its receptors contribute to CF pathogenesis.

### Liver fibrosis

Fibrosis is a frequent, life-threatening complication of most chronic liver diseases [55]. Despite recent achievements in the understanding of the pathogenesis, the translation of this knowledge into clinical application is still limited. Liver fibrosis can result from persistent liver jury, including alcohol abuse, viral hepatitis, metabolic diseases, and cholestatic liver diseases [56]. It is the hallmark feature associated with portal hypertension and liver failure, and the risk of hepatocellular carcinoma [57,58]. Hepatic stellate cells (HSCs), which are located in the space of Disse between hepatocytes and sinusoidal endothelium, play a central role in the progression of ECM deposition and liver fibrosis by the transformation of HSCs into proliferative and fibrogenic myofibroblast-like cells [58].

A recent study has confirmed that HMGB1 up-regulates alpha-smooth muscle actin (α-SMA) expression and suppresses the collagen-degrading matrix metalloproteinases-2 (MMP-2) activity in HSCs [59]. Moreover, it activates HSCs and exhibits pro-fibrogenic effects on liver grafts either by increasing the HSC population and ECM content in liver grafts, or by transforming HSCs into myofibroblasts. The serum level of HMGB1 is significantly higher in patients with low liver fibrosis (fibrosis score 1–2) compared to those with high liver fibrosis (fibrosis score 3–4), which is a non-invasive, repeatable, and convenient marker for distinguishing advanced fibrosis from low fibrosis in chronic hepatitis B virus patients [60]. Another research has shown that HMGB1 is up-regulated during liver fibrosis in rats and its expression is closely related to collagen deposition, while inhibition of HMGB1 expression by small interfering RNA (siRNA) inhibits the synthesis of α-SMA and collagen in transfected HSCs [61]. In the study of the treatment of liver fibrosis, curcumin is effective in preventing liver fibrosis partly because of reducing TLR2, TLR4 and HMGB1 expression by inhibiting pro-inflammatory mediators and HSCs activation [62]. *In vitro*, HMGB1 can significantly stimulate migration of HSCs, while TLR4-neutralizing antibody inhibits HMGB1-enhanced phosphorylation of jun N-terminal kinase (JNK) and phosphatidylinositol 3-kinase (PI3K)-serine/threonine kinase (Akt) and activation of NF-κB, suggesting TLR4-dependent signal pathways are involved in the HMGB1-induced proliferation, migration and pro-fibrotic effects of HSCs [63].

These data further indicate a significant pro-fibrotic function of HMGB1 and inhibiting HMGB1 expression or its related signal such as HMGB1/TLR4 might be a potential strategy to treat liver fibrosis.

### Renal fibrosis

Tissue fibrosis is a hallmark of a variety of chronically failing organs, including progressive renal disease [3]. Renal fibrosis is defined by the accumulation of interstitial leucocytes and myofibroblasts that contribute to abnormal accumulation of ECM and eventual tubular atrophy and loss of renal function [64]. It is considered to be the common final pathway by which kidney diseases with variable aetiology progresses to end-stage renal failure and therefore important to identify factors that participate in the initiation of tubulointerstitial inflammation and subsequent interstitial fibrosis during progressive renal injury. It has been demonstrated that some environmental factors such as passive smoking can lead to renal fibrosis [65]. Moreover, it can also been driven by transforming growth factor-beta (TGF-β) family of cytokines, connective tissue growth factor, NF-κB, Notch, and other factors such as HMGB1 [66,67].

Data have shown that recombinant human HMGB1 induces alterations in epithelial morphology consistent with epithelial–mesenchymal transition (EMT) including reducing E-cadherin expression, increasing α-SMA expression and enhancing cell migration in human proximal tubular epithelial cells [67]. Inhibition of RAGE and TGF-β1 by using their neutralizing antibodies confers a protective effect against the alterations induced by HMGB1, suggesting their involvement in mediating HMGB1 actions [67]. In patients with chronic renal injury, TLR2 is markedly up-regulated on tubular and tubulointerstitial cells, while renal injury is associated with a marked up-regulation and change in distribution of TLR2, up-regulation of murine TLR2 danger ligands Gp96, biglycan, and HMGB1 in mice with obstructive nephropathy [64]. However, the absence of TLR2 does not affect the development of chronic renal injury and fibrosis.

It reveals that TLR2 initiates a renal inflammatory response during obstructive nephropathy, but does not play a significant role in the development of renal fibrosis. On the other hand, it is likely that some pro-fibrotic factors such as HMGB1 or HMGB1/TLR2 inhibition may be more important than TLR2 alone inhibition in interfering with renal fibrosis.

### Pulmonary fibrosis

PF is a chronic interstitial lung disease characterized by acute and chronic inflammation, vascular leakage and myofibroblast recruitment that leads to an irreversible process [68,69]. Following lung damage, the accumulated myofibroblasts activate and secrete ECM, and form fibrotic foci [69]. In addition to the poor prognosis, the pathogenesis of PF remains to be elucidated and no effective therapeutic strategy has been established.

A recent study provides evidence that bleomycin administration to wild-type mice induced HMGB1 production from inflammatory cells that accumulated within the air space, while RAGE deficiency was accompanied by dampened levels of TGF-β and PDGF [70]. Coculture with HMGB1 induces EMT in AECII from wild-type mice other than those derived from RAGE^−/−^ mice, such that the protective effects are possibly because of an inability of HMGB1, a well-characterized RAGE ligand, to contribute to fibrosis through EMT and pro-fibrotic cytokine production [70]. However, blocking RAGE signalling *via* administration of soluble RAGE (sRAGE), a decoy receptor, is unable to ameliorate bleomycin-induced PF in C57Bl/6 mice [71]. In PF patients, HMGB1 levels in bronchoalveolar lavage fluid (BALF) and in the lungs are significantly increased compared with control cases, and it is predominantly expressed in alveolar macrophages, infiltrating inflammatory cells, and AEC in lung tissues from patients [72]. In addition, HMGB1 can directly stimulate fibroblast proliferation *in vitro*; in mice, anti-HMGB1 antibody or ethyl pyruvate protects mice from bleomycin-induced PF through attenuating inflammation, apoptosis and fibrosis [72]. Another study demonstrates that the HMGB1 released by wounded epithelial cell monolayers, accelerates wound closure in the distal lung epithelium *via* the activation of TGF-β1, indicating that the complete inhibition of this response by anti-inflammatory agents may have adverse effect on the lung epithelial repair [73]. If this inflammatory response becomes uncontrolled and maladaptive because of too severe or repeated insults, it may become an important mechanism in the development of PF. Thus, further investigation of the interplay between HMGB1 signal and TGF-β1 pathway may provide new insights for more clear understanding of the related diseases and therapies such as PF.

Beyond that, further research has reported that HMGB1 is increased in the lungs of PF patients after acute exacerbation and that the alveolar capillary augmented lesions with reduced level of thrombomodulin, an intrinsic inhibitor of HMGB1, may exacerbate alveolar damage and fibrogenesis [74]. Interestingly, polymyxin B (PMX), which is originally developed for the removal of endotoxin and treatment of endotoxemia, has been reported to reduce the serum HMGB1 levels in PF patients with acute exacerbation and that HMGB1 is detected in washing medium from PMX fibre, suggesting that PMX may directly absorb HMGB1 and it may be an efficacious therapeutic option for acute exacerbation in PF [75]. These results make it clear that the inhibition of HMGB1 such as PMX or thrombomodulin may clinically be effective for the treatment of PF.

### Myocardial fibrosis

Myocardial fibrosis, characterized by over-accumulation of fibroblasts and deposition of increasing amounts of ECM proteins in the myocardium, is defined as a key component of heart failure [76]. Up to now, the molecular mechanisms underlying cardiac fibrosis are not clear, and factors contributing to the cardiac dysfunction remain to be elucidated. But inhibiting the abnormal proliferation and activity of cardiac fibroblasts and evade maladaptive processes may represent a potential strategy to attenuate myocardial fibrosis such as the cardiac protective effect of 5-azacytidine in modulating cardiac fibroblasts [77].

A recent *in vivo* study reveals that HMGB1 is diffusely expressed in the myocardium, while HMGB1 silencing ameliorates LV dysfunction and remodelling, and decreases collagen deposition in diabetic mice [78]. *In vitro*, high glucose (HG) can induce HMGB1 translocation and secretion in both viable cardiomyocytes and fibroblasts. Administration of HMGB1 dose-dependently increases the expression of collagens I and III, and TGF-β1, while pharmacological (neutralizing anti-HMGB1 antibody) or genetic (shRNA-HMGB1) inhibition of HMGB1 in cardiac fibroblasts reduces HG-induced collagen production, MMPs activities, proliferation, and activates MAPK signalling [78]. Given the cardio-protective effects of HMGB1 silencing, inhibition of HMGB1 may improve myocardial fibrosis and undoubtedly provide new target for its intervention.

## Targeting HMGB1 as therapy in fibrotic diseases

**Fig. 2:**
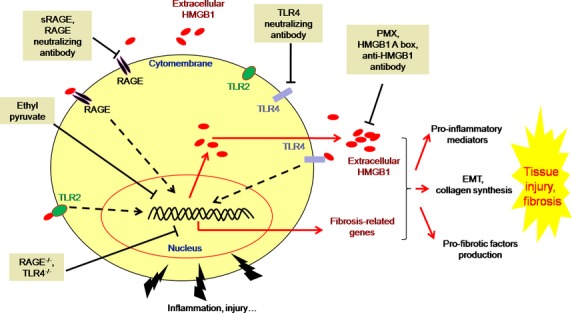
Agents targeting HMGB1 or its receptors in the studies of tissue fibrosis. In addition to the inhibition of HMGB1 or its receptors by neutralizing antibodies [54,63,67,78], Antagonistic HMGB1 treatment, based on HMGB1 silencing [61,78], RAGE or TLRs knockout [54,64,72], small-molecule inhibitors such as ethyl pyruvate [72], has proven successful in a wide range of experiments, resulting in reduced severity of fibrotic models and decreased lethality, all of which represent therapeutic measures for blocking HMGB1.

Emerging evidence indicates that HMGB1, a pluripotent mediator, contributes to many fibrotic diseases such as CF, liver fibrosis and progressive PF, indicating that HMGB1 may be a promising therapeutic target for such diseases. Moreover, agents targeting HMGB1 have shown successful *in vivo* and *in vitro* studies (Fig. 2). In addition to the inhibition of HMGB1 or its receptors by neutralizing antibodies [54,63,67,78], more researchers are carried out to elucidate different mechanisms involved in HMGB1 release and signalling, and illustrate different methods of suppressing its release, or the damage that is caused. Antagonistic HMGB1 signals, based on HMGB1 silencing [61,78], RAGE or TLRs knockout [54,64,70], small-molecule inhibitors such as ethyl pyruvate [60], have proven successful in a wide range of experiments, resulting in reduced severity of fibrotic models and decreased lethality, suggesting that HMGB1 may be a therapeutic target for fibrotic diseases.

## Conclusions and future prospects

As reported, the epithelial cells that line the surface of a tissue or organ and mesenchymal cells such as fibroblasts are two very common cell types in any given animal models [79]. Similarly, epithelial cells and mesenchymal cells are essential cell types involving tissue fibrosis. As the fibrotic diseases are partly because of the progressive injury of epithelial cell and excessive proliferation of (myo)fibroblasts, with which the normal tissue structure is destructed and the production of ECM is increased. Thus, it may provide new insights into the treatment of tissue fibrosis by reversing or inhibiting the abnormal changes of cell types if possible. Surprisingly, emerging experimental evidence indicates that HMGB1 plays an important role in EMT, a change in cell types in renal and lung [55,58], while HMGB1 signal blockade reveals inhibited EMT, suggesting that HMGB1 may elicit the similar effect in other organs. As such, the investigations of HMGB1 inhibition may yield detailed molecular insights into cell fate decisions for the switching between epithelial and ECM-produced cells. In addition, the polyceptor interaction with RAGE and TLRs, and its feedforward mechanistic nature, plays a key role in the pathogenesis of fibrogenesis, resulting in damage to the vital organs. However, it has been proven that inhibiting HMGB1 activity or its related signal such as HMGB1/TLR4 and HMGB1/RAGE is beneficial to treat fibrotic diseases *via* suppressing EMT process, tissue injury, collagen synthesis and pro-fibrotic cytokines production, indicating HMGB1 signals significantly mediate the progress of fibrogenesis.

Although intervention with HMGB1 shows remarkable effects in ameliorating the pathogenic condition and attenuating disease progression in pre-clinical researches, the clinical using HMGB1-specific antagonists have not been performed in fibrotic patients. In addition, of note, if developing HMGB1 inhibitors for use in clinical, safety will be a crucial issue, with concerns associated with the complexity of HMGB1 biology and even competing functions of extracellular HMGB1 as it interacts with multiple signalling systems. Furthermore, dynamic post-translational modifications of HMGB1 might shift its activity from injury to tissue repair. In the future, studies will further define the immunological properties of HMGB1 and optimize strategies for blocking its abnormal activation in fibrotic diseases. Because the amino acid sequence of HMGB1 is highly conserved in all mammals, rapid translocation of antagonists developed in animals to the setting of human fibrotic diseases should be hopeful and possibly a new approach. Therefore, the clinical application of HMGB1 inhibitors in treating fibrotic patients is full of prospects, and the study of HMGB1 and its signalling pathways continues to increase the possibilities in drug design and therapy to prevent and/or cure the refractory diseases.
